# Public-private mix in health systems and repercussions for health inequalities in Latin American countries: A scoping review protocol

**DOI:** 10.1371/journal.pone.0305437

**Published:** 2026-02-19

**Authors:** Eduarda Ferreira dos Anjos, Suelen Carlos de Oliveira, Adriana Mendoza-Ruiz, Maria del Pilar Flores-Quispe, Adelyne Maria Mendes Pereira, Carl Kendall, Ligia Regina Franco Sansigolo Kerr, Naiá Ortelan, Elzo Pereira Pinto Junior, Natalia Romero, Alastair Leyland, Mauricio Lima Barreto, Cristiani Vieira Machado, on behalf of the Unit on the Social and, Environmental Determinants of Health Inequalities (SEDHI)

**Affiliations:** 1 National School of Public Health (ENSP), Oswaldo Cruz Foundation, Rio de Janeiro, Rio de Janeiro, Brazil; 2 Centre for Data and Knowledge Integration for Health (CIDACS), Oswaldo Cruz Foundation, Salvador, Bahia, Brazil; 3 School of Medicine, Federal University of Ceará (UFC), Fortaleza, Ceará, Brazil; 4 Universidad Internacional del Ecuador (UIDE), Quito, Pichincha, Ecuador; 5 MRC/CSO Social & Public Health Sciences Unit, University of Glasgow, Glasgow, United Kingdom; Universidade Federal de Minas Gerais, BRAZIL

## Abstract

**Introduction:**

Health system models in Latin American countries express differences in the state’s role in regulating, financing and providing health services, as well as in the coexistence of different care arrangements involving public and private entities. This scoping review will seek to identify evidence on how the different public-private configurations of health systems influence health inequalities in Latin America.

**Methods and analysis:**

This protocol will be guided by the scoping review methodology developed by the Joanna Briggs Institute. The results will be presented according to the PRISMA-ScR protocol. Searches will be carried out on the Scielo, Lilacs, Embase, Pubmed, Web of Science and Scopus databases. The inclusion criteria will be publications whose central theme is the public-private mix of health systems in Latin America between January 2000 and March 2024. Exclusion criteria will be clinical trials, incomplete studies or in the design phase, duplicate publications, pre-print studies and gray literature (interim reports, unpublished texts, dissertations, and theses. The steps for carrying out the scope review will: (1) identifying the research question; (2) identifying relevant studies; (3) study selection; (4) charting the data; and (5) collating, summarizing, and reporting the results. Two independent reviewers will select the articles. The results will be described, analyzed, and categorized through narrative synthesis, correlating with the research objectives and questions.

**Expected results:**

It is hoped that this scoping protocol will provide a comprehensive framework for investigating the gaps in the current literature on the public-private mix in Latin America and the effects on health inequalities. **Link to the protocol record in the Open Science Framework (OSF):** https://osf.io/rkzx3/?view_only=4594a2c52f2d47128d805fdd9dd1359b.

## Introduction

Health systems in Latin American countries have been structured in various ways throughout the 20th century, generally involving a strand of collective public health actions and another of medical care aimed at workers in the formal labour market. Throughout their history, the consolidation of these systems has been hampered by economic restrictions, democratic instability, social inequality, and widespread informality in the labour market [1]. The diversity of ethnic and cultural characteristics, the population’s lifestyles (urban, rural, and remote rural) and the socioeconomic level of the population in the different nations of Latin America add to the challenges for health systems [2].

Reforms adopted over the years in various regional countries expanded the private health sector under state incentives associated with new business dynamics, resulting in a complex public-private mix [3–5]. This complexity is expressed in the segmentation of the population according to their affiliation to services, the composition of the supply of care, health spending, the diversity of providers working in the sector, the multiplicity of health professionals’ ties, as well as the different coverage and access between groups according to their ability to pay or pay directly for services [2,6,7].

In addition, there are historical-structural challenges in the organization, financing, and provision of health systems in Latin America, which have recently been highlighted by the COVID-19 pandemic, which has exacerbated the inequalities and weaknesses of these systems, revealing their limitations and potential [8–10]. In this sense, understanding public-private health arrangements in Latin America over the last few decades and considering the impacts of the recent pandemic can help direct public policies with a focus on state-market relations. Thus, this protocol proposes a scoping review to identify and explore the evidence on the effects of the public-private mix of health systems on health inequalities in Latin America.

## Methods and analysis

The review will cover the period from 2000 to 2024. The time frame is justified because it covers more than two decades marked by changes in various health systems in Latin America but with persistent health inequalities. The scoping review technique was selected for its potential in mapping complex and heterogeneous literature, considering the particular contexts of Latin American countries, as well as being useful for providing decision-makers with information on how the topic has developed over time, as well as gaps and the need to strengthen public policy agendas.

The construction of this protocol was based on internationally recognized and validated scoping review guidelines, as well as articles in the field of public health that have adopted this methodological design. This review follows the Joanna Briggs Institute (JBI) and the Preferred Reporting Items for Systematic Reviews and Meta-Analyses Extension for Scoping Reviews (PRISMA-ScR) and your checklist, will follow the following methodological steps: (1) identifying the research question; (2) identifying relevant studies; (3) study selection; (4) charting the data; and (5) collating, summarizing and reporting the results.

This protocol was registered on the Open Science Framework platform following the JBI recommendation, to conduct an impartial review, avoid duplication of research, and guarantee the originality of the work. It can be consulted via the register: https://osf.io/rkzx3/?view_only=4594a2c52f2d47128d805fdd9dd1359b.

### Step 1: Identifying the research question

Public-private relations in health systems and their implications for health inequalities in Latin America are the focus of this review. In particular, the participation and arrangements between the public and private sectors in the financing, coverage, and provision of services will be highlighted. The focus, question and hypothesis of this work are detailed in Box 1.

**Box 1. Focus, question, and hypothesis of this scoping review.**

**Focus**

Public-private mix and health inequalities.

**Question**

What are the effects of the public-private mix of health systems on health inequalities in Latin America?

**Hypothesis**

The public-private mix of health systems, expressed in the conditions of financing, coverage, and provision of services, influences health inequalities in Latin America.

Source: Elaborated by the authors.

The construction of the research questions was based on the PCC mnemonic, where ‘P’ represents the population, ‘C’ the concept and ‘C’ the context. The ‘population’ was taken to be the equivalent of the unit of analysis for public health (in this case, “Health Systems”). For the context, in addition to the term Latin America, seven countries were included in the bibliographic search: Argentina, Brazil, Chile, Colombia, Ecuador, Mexico and Peru (Box 2).

**Box 2. Population (P), Concept (C) and Context (C).**

**Population (P): Health Systems**

**Concept (C).**

**Health Systems.**

“A set of political, economic and institutional relationships responsible for conducting the processes related to the health of a given population, which take the form of organizations, rules and services aimed at achieving results consistent with the concept of health that prevails in society” [11].

**Public-private mix.**

“Public and private designate different situations of more complex social groups and refer, respectively, to what belongs to the collective, to the community and to individual members of a given society. The ownership of a given asset dictates the nuances of the public/private dichotomy. However, when the asset is health, categorizing public and private is admittedly difficult. There is widespread recognition of the complexity involved in defining the collective and the individual in the health-disease process. Thus, the public-private dyad more accurately serves the classification of institutionalized responses and health problems than the automatic transposition of the meaning of collective or individual ownership to events related to the care of diseases and illnesses or risk prevention. It is the institutions organized to respond to health demands and needs that, in general, receive the public or private service offerings” [12].

**Health inequalities.**

“Health inequalities are the systematic, avoidable and unfair differences in health outcomes that can be observed between populations, between social groups within the same population or as a gradient across a population ranked by social position” [13].

**Context (C).** Latin America (Argentina, Brazil, Chile, Colombia, Ecuador, Mexico and Peru).

Source: Elaborated by the authors.

The seven countries were selected because they are populous and concentrate most of the region’s publications, as well as because they meet criteria of interest for the analysis, such as the persistence of significant socioeconomic inequalities and the presence of varied health system arrangements, including the universal model, the social insurance model, and different private sector financing and provision models.

The health systems of the seven countries selected for the study are briefly described in Box 3, covering coverage, regulation, financing and system organization. Of these, three are federal countries (Argentina, Mexico and Brazil) and four are unitary republics (Chile, Colombia, Peru and Ecuador).

**Box 3. General characteristics of Health Systems in seven countries of Latin America.**

**Federative Republics**

**Argentina**

The health system in Argentina is based on a federal political structure, decentralized and organized into three distinct sub-sectors: (i) public sub-sector, financed by taxes and the national, provincial and municipal budgets, responsible for covering approximately 43.1% of the population; (ii) the social insurance subsystem, financed by contributions from formal market workers (3% of salary) and employers (5%), which covers the majority of the Argentine population (around 62.7%); e (iii) the private subsystem, used by approximately 7 million individuals, which includes health plans or voluntary insurance companies and is characterized by the pre-payment of the medical services provided [14].

**Mexico**

Mexico has a health system comprising both public and private services. The three main components are as follows: (i) social security for workers, which serves most of the population mainly through the Instituto Mexicano de Seguro Social (IMSS in Spanish) and the Instituto de Seguridad y Servicios Sociales de los Trabajadores del Estado (ISSSTE in Spanish); (ii) the public assistance services for the population not covered by social security. These consist of state services and/or Popular Health Insurance (2004–2018), which was replaced first by the Health Institute for Wellbeing (INSABI) (2019–2022) and then, from 2023, by the Mexican Welfare Institute (IMSS-BIENESTAR); and (iii) a private sector comprising insurance companies and service providers with no links to the public sector.  [15]. In 2018, a reform of the health system began, culminating in the extinction of the Seguro Popular de Salud and the creation of the Instituto de Salud para el Bienestar (INSABI in Spanish) to serve the population not covered by social security institutes. The restructuring proposal was based on a centralized federal organization and the integration of the financing and provision of public services in the health system and a reduction in private participation [16]. In 2023, however, the government proposed the extinction of INSABI, so that its attributions could be taken over by IMSS-BIENESTAR (in Spanish).

**Brazil**

Taxes and social contributions fund the Brazilian Sistema Único de Saúde (SUS in Portuguese). However, underfunding is a major obstacle facing the system. It significantly depends on the private sector for beds, laboratories, specialized services, and diagnostics. Equally contradictory is the marked presence of private plans and insurance paid for by around 25% of the Brazilian population who also have access to the SUS [6]. Although the private sector is constitutionally considered complementary, creating a universal, public, and free healthcare system, based on the principles of decentralization, comprehensiveness, and community participation, has not been enough to curb the persistence and even expansion of the private sector in Brazilian healthcare [17,18].

**Unitary Republics**

**Chile**

Since the reform of the 1980s, which eliminated previously existing social solidarity mechanisms, Chile has had a dual health system, where the public sector is represented by Fondo Nacional de Salud (Fonasa in Spanish) and the private sector by Instituiciones de Salud Previsional (Isapres in Spanish). It is characterized by the prioritization of markets in healthcare, through a public-private mix in regulation, financing, and service provision [19]. It is recognized as one of the most fragmented and segmented in Latin America [20–22]. Chile’s re-democratization failed to reverse this model. The country is currently discussing a proposal to reform the system based on the right to health, equity and access to health services [23].

**Colombia**

The 1993 reform created the Sistema General de Seguridad Social en Salud (SGSSS in Spanish). It implemented the so-called Structured Pluralism model, characterized as a regulated private health insurance sector, based on competition between public and private agents, aiming for greater efficiency in financing the system. The reform institutionalized the role of private agents in social insurance, with significant growth in these agents [24]. Insurance is compulsory through the Entidades Promotoras de Salud (EPS in Spanish), which can be public or private. There are two regimes: Contributory, which offers benefits to people who can pay, and Subsidized, which provides a package of essential services with state funding for low-income people. The existence of different regimes, with different “targeted basket of services”, has resulted in segmentation of the population and barriers to access the care of the poorest, which have not been overcome even with the adoption of the equalization of the “basket of services” in the last decade [25–27]. In 2023, a reform proposal was launched, which is still underway, to reduce inequalities in service provision and access.

**Peru**

The Peruvian health system is based on a public-private mix, structured into two distinct subsystems. The public subsystem is divided into two regimes: direct contributory, corresponding to social insurance for formal workers; and subsidized contributory, made up of the Ministry of Health and the Seguro Integral de Salud (SIS in Spanish), which acts as an autonomous public insurer [28]. The private subsystem, subdivided into for-profit and non-profit institutions, includes Entidades Prestadoras de Salud (EPS in Spanish), private insurers, clinics, and civil society organizations. Most non-profit institutions provide primary care services and often receive financial resources from external donors, internal donors, the government, and families [29]. The health system is characterized by segmentation, that is, the absence or low level of integration between different sub-sectors and functions, an aspect found in other countries in the region [27,30].

**Ecuador**

The country has a system based on a public-private mix. At the public level, there are social insurance institutions aimed at maintaining the workforce and serving the low-income population, such as the Ministry of Health and the Instituto Ecuatoriano de Seguridad Social (IESS in Spanish), which serve very heterogeneous social sectors [31]. There are also social insurance institutions dedicated to the health care of the armed forces, police officers and their families, such as the Instituto de Seguridad Social de las Fuerzas Armadas (ISSFA in Spanish) and the Instituto de Seguridad Social de la Policía Nacional (ISSPOL in Spanish). The private sector includes for-profit and non-profit entities [32,33]. Private insurance companies and pre-paid drug plans cover approximately 3% of the middle and upper-class population. One of the main challenges facing the health system is the high rate of out-of-pocket spending, which in 2020 reached 33.8% of the total health bill [34].

Source: Elaborated by the authors.

The seven countries show different levels of socioeconomic and health inequalities. The data related to the Gini coefficient reiterates the socioeconomic disparities in Latin American countries. In 2020, the average coefficient was 0.46 for the entire region. Ecuador (0.46) and Peru (0.46) have indicators similar to or close to the regional average, making it clear that they face different gradients of inequalities in terms of health and socioeconomic conditions. Mexico (0.45) showed the smallest difference between the richest and poorest sections of its population. In contrast, Colombia (0.55), Brazil (0.51) and Chile (0.47) stood out for their greater concentration of income, making them among the most unequal countries in the world [35].

The Latin American federations, Brazil represents the case of a universal health system, based on a comprehensive conception of social security, with many differences in access to health between regions of the country and a strong presence of the private sector; while Mexico has a social insurance system, with high private spending through direct disbursement by families. The Argentine system has maintained its corporate base, with the presence of the private sector in the provision of services and the existence of specific public programs [6].

Chile and Colombia are illustrative cases of neoliberal reforms that radically altered the role of the state and markets in health. In Chile, the reform began at the end of the 1970s under the military dictatorship, resulting in a dual system. In Colombia, in the 1990s, under a democratic regime, the Structured Pluralism model was implemented [19,24]. Ecuador and Peru are characterized by mixed systems, with a weakened public sector compared to the private sector [27,29–33].

### Step 2: Identifying relevant studies

#### Data sources.

The search for scientific articles will be carried out in six databases: Lilacs via the Biblioteca Virtual de Saúde (BVS in Portuguese); Embase; Pubmed; Scielo; Web of Science; and Scopus. Access to the Web of Science and Scopus databases will be via the Portal de Periódicos Capes (in Portuguese). In addition, references relevant to the topic will be included in the articles found.

### Search strategy

The search will be carried out using a main search strategy which will be adapted for each database according to the PCC and the defined inclusion and exclusion criteria. The search strategies for the three databases with controlled vocabulary, Lilacs/BVS, Embase and Pubmed, will use the English language descriptors indexed: Descriptors in Health Sciences (Decs in Portuguese), Emtree term and Medical Subject Headings (MESH), respectively (Box 4). In the other databases, the strategy will exclusively use keywords (S1 Appendix).

**Box 4. Search terms.**

1. “Health system” OR “Health systems” OR “Health care system” OR “Health care systems” OR “Health policy” OR “Health polic*” OR “Health reform*”
2. “Public private” OR Public-private OR “Public and private” OR “Private sector” OR Privatization OR “Private plans” OR “Private insurance”
3. Financ* OR Spending OR Expense* OR Expenditures* OR Costs OR Spending OR Payment* OR Coverage OR Deliver* Or Provider* OR Regulation OR Access OR Utilization OR Provision
4. Latin America OR Brasil* OR Brazil OR Equador OR Ecuador OR México OR Mexico OR Chile OR Colômbia OR Colombia OR Peru OR Per* OR Argentin*.
5. “Health inequalities” OR “Health disparities” OR “Health assymetries” OR “Health inequities”

Source: Elaborated by the authors.

### Step 3: Study selection

After searching in the descriptor and/or keyword fields (depending on the database), the texts will first be screened by reading the title and abstract and then by reading the full text.

The selection of the documents to be included in this scope review at each stage will be carried out by two reviewers independently. Any disagreements will be resolved by consensus or by decision of a third reviewer. To increase uniformity and accuracy, all reviewers will first carry out a calibration exercise. Subsequently, reviewers in pairs will independently analyze all the records using the Rayyan platform (https://www.rayyan.ai/). Full articles that meet the inclusion criteria established for the study will be selected and examined in detail.

Monitoring of the study selection process will be detailed in a flowchart (according to PRISMA-ScR guidelines), according to the identification, selection, eligibility, and inclusion stages. The search results will be managed using the ‘Zotero’ software to collect, store and organize the references. Using the reference manager will allow for organization into different groups, facilitating sharing with the researchers participating in this study.

### Inclusion criteria

This review will consider publications whose central theme is the public-private mix of health systems in Latin America (especially those dealing with Brazil, Ecuador, Mexico, Chile, Colombia, Peru and Argentina, individually or comparatively with other countries), published between January 2000 and March 2024.

The identification and selection of relevant publications will include the following types of documents: essays, qualitative and quantitative research (case studies, ecological, cross-sectional and time series studies), analysis and evaluation of health policies and systems, published in English, Portuguese, and Spanish.

Only open-access scientific articles published in journals indexed in the aforementioned databases will be included, as they undergo peer review. Theses and dissertations will not be included (since their end product is usually scientific articles published in journals) and books or chapters (the databases selected are incomplete for such works, and not all are peer-reviewed and open access).

### Exclusion criteria

Clinical trials, incomplete studies or in the design phase, duplicate publications, pre-print studies, systematic reviews, narrative reviews and gray literature (interim reports, unpublished texts, dissertations and theses) will be excluded.

### Step 4: Charting the data

The search strategy will be carried out in three stages: (1) Initial search in two databases; (2) Identification of terms to be included, enrichment of the search strategy and search in the other databases; and (3) Review of references of included documents to track down and evaluate the inclusion of other works. This strategy will be guided by a data extraction form developed electronically to extract the information, which will be filled in independently by two researchers and then converted into a file with a format compatible with the Microsoft Excel® program (S2 Appendix). The file generated will be the database to be used in the subsequent phase of this review and will contain information identifying the study (title, first author, year, objectives, study design, funding) and data related to the research questions (geographical context/countries).

In this way, the following information will be mapped by the reviewers: characteristics and details of the study (title, first author, year, country, objectives and study design) and data related to the research questions (public-private configuration in financing, coverage and provision of services, as well as repercussions for health inequalities and response to covid-19).

### Step 5: Collating, summarizing and reporting the results

A descriptive analysis of the included studies will be carried out using the concepts described in the form used to extract the data and other characteristics relevant to the findings. The content of the findings will be categorized into “financing models”, “service coverage”, “public-private mix in the provision of services” and “repercussions on health inequalities”.

The results will be summarized to return to the research question and the proposed objective. The scoping review by Nyanchoka et al [36] will be used as a reference to choose the appropriate resources for presenting the results. The results will be presented in graphs with information on the year of publication, language, countries studied and dimensions of analysis, for a general characterization of the studies included in the review, as well as tables containing the results found by country for each dimension of analysis (financing, coverage and provision), allowing for an overview of the information from each country.

The plan is to hold a meeting with experts and stakhoders on the subject (managers and representatives of social participation in health) to present and discuss the results, before writing the final review article. The work will proceed according to the detailed plan in S3 Appendix.

The supplementary materials for this manuscript contain details of the search strategy (S1 Appendix), the data extraction instrument (S2 Appendix), and the timeline for the review (S3 Appendix) for this protocol article.

## Supporting information

S1 Appendix Search strategy. (DOCX)

S2 Appendix Data extraction instrument. (DOCX)

S3 Appendix Timeline for the review. (DOCX)

S1 Table PRISMA-ScR checklist. (DOCX)

### Disseminations of Knowledge

This scoping protocol will provide a comprehensive framework for investigating the gaps in the current literature on the public-private mix in Latin America and the effects on health inequalities, guided by the guidelines recommended by the Joanna Briggs Institute and the PRISMA-ScR protocol. This evaluation will provide tools and evidence to policymakers of the public-private mix in Latin America and the effects on health inequalities. We will disseminate the data in scientific journals, reports, and policy briefings targeting policymakers and civil society.
