# Supplementary material for: Public-private mix in health systems and repercussions for health inequalities in Latin American countries: A scoping review protocol
Source: PLoS One. 2026 Feb 19;21(2):e0305437. doi: 10.1371/journal.pone.0305437 (PMC12919785; doi:10.1371/journal.pone.0305437)
Supplement: S1 Appendix — (DOCX) [file pone.0305437.s001.docx]

**S1 Appendix** **1**– **Search strategy**

| **BVS** |  |
| --- | --- |
| **Strategy:** ("Health system" OR "Health systems" OR "Health care system" OR "Health care systems" OR "Health policy" OR "Health polic*" OR "Health reform*" OR "Unified Health System" OR sus OR "National Health Systems") AND (("Public private" OR public-private OR "Public and private" OR "Private sector" OR privatization OR "Private plans" OR "Private insurance" OR "Public Sector" OR "Public-Private Sector Partnerships" OR "Public-Private Mix") OR (financ* OR spending OR expense* OR expenditures* OR costs OR spending OR payment* OR coverage OR deliver* OR provider* OR regulation OR access OR utilization OR provision OR "Health Consortia" OR "Healthcare Financing" OR "Health Insurance")) AND ("Latin America" OR "Latinoamérica" OR "América Latina" OR brasil* OR brazil OR equador OR ecuador OR méxico OR mexico OR chile OR colômbia OR colombia OR peru OR perú OR peru* OR argentin*) AND ("Health inequalit*" OR "Health inequit*" OR "Health disparit*" OR "Health assymetr*" OR "Health Status Disparities" OR "Socioeconomic Disparities in Health" OR "Social Determinants of Health") AND (year_cluster:[2000 TO 2022]) | |
| **Fields used in the database:**   - Title; - Subject; - Abstract; - Period: 2000-2024 | |
| **Embase** |  |
| **Strategy:** ('health system':ti,ab,kw OR 'health systems':ti,ab,kw OR 'health care system':ti,ab,kw OR 'health care systems':ti,ab,kw OR 'health policy':ti,ab,kw OR 'health polic*':ti,ab,kw OR 'health reform*':ti,ab,kw OR 'unified health system':ti,ab,kw OR sus:ti,ab,kw OR 'national health systems':ti,ab,kw) AND (('public private':ti,ab,kw OR public:ti,ab,kw) AND private:ti,ab,kw OR 'private sector':ti,ab,kw OR privatization:ti,ab,kw OR 'private plans':ti,ab,kw OR 'private insurance':ti,ab,kw OR 'public sector':ti,ab,kw OR 'public-private sector partnerships':ti,ab,kw OR 'public-private mix':ti,ab,kw OR financ*:ti,ab,kw OR expense*:ti,ab,kw OR expenditures*:ti,ab,kw OR costs:ti,ab,kw OR spending:ti,ab,kw OR payment*:ti,ab,kw OR coverage:ti,ab,kw OR deliver*:ti,ab,kw OR provider*:ti,ab,kw OR regulation:ti,ab,kw OR access:ti,ab,kw OR utilization:ti,ab,kw OR provision:ti,ab,kw OR 'health consortia':ti,ab,kw OR 'healthcare financing':ti,ab,kw OR 'health insurance':ti,ab,kw) AND ('latin america':ti,ab,kw OR 'latinoamérica':ti,ab,kw OR 'américa latina':ti,ab,kw OR brasil*:ti,ab,kw OR brazil:ti,ab,kw OR equador:ti,ab,kw OR ecuador:ti,ab,kw OR méxico:ti,ab,kw OR mexico:ti,ab,kw OR chile:ti,ab,kw OR colômbia:ti,ab,kw OR colombia:ti,ab,kw OR peru:ti,ab,kw OR perú:ti,ab,kw OR peru*:ti,ab,kw OR argentin*:ti,ab,kw) AND ('health inequalit*':ti,ab,kw OR 'health inequit*':ti,ab,kw OR 'health disparit*':ti,ab,kw OR 'health assymetr*':ti,ab,kw OR 'health status disparities':ti,ab,kw OR 'socioeconomic disparities in health':ti,ab,kw OR 'social determinants of health':ti,ab,kw) AND [2000-2022]/py | |
| **Fields used in the database:**   - Title; - Subject; - Abstract; - Period: 2000-2024 | |
| **PubMed** |  |
| **Strategy:** ("Health system" OR "Health systems" OR "Health care system" OR "Health care systems" OR "Health policy" OR "Health polic*" OR "Health reform*" OR "Unified Health System" OR SUS OR "National Health Systems") AND (("Public private" OR Public-private OR "Public and private" OR "Private sector" OR Privatization OR "Private plans" OR "Private insurance" OR "Public Sector" OR "Public-Private Sector Partnerships" OR "Public-Private Mix") OR (Financ* OR Spending OR Expense* OR Expenditures* OR Costs OR Spending OR Payment* OR Coverage OR Deliver* OR Provider* OR Regulation OR Access OR Utilization OR Provision OR "Health Consortia" OR "Healthcare Financing" OR "Health Insurance")) AND ("Latin America" OR "Latinoamérica" OR "América Latina" OR Brasil* OR Brazil OR Equador OR Ecuador OR México OR Mexico OR Chile OR Colômbia OR Colombia OR Peru OR Perú OR Peru* OR Argentin*) AND ("Health inequalit*" OR "Health inequit*" OR "Health disparit*" OR "Health assymetr*" OR "Health Status Disparities" OR "Socioeconomic Disparities in Health" OR "Social Determinants of Health") | |
| **Fields used in the database:**   - All Fields; - Period: 2000-2024 | |
| **Scielo** |  |
| **Strategy:** ("Health system" OR "Health systems" OR "Health care system" OR "Health care systems" OR "Health policy" OR "Health polic*" OR "Health reform*" OR "Unified Health System" OR SUS OR "National Health Systems") AND (("Public private" OR Public-private OR "Public and private" OR "Private sector" OR Privatization OR "Private plans" OR "Private insurance" OR "Public Sector" OR "Public-Private Sector Partnerships" OR "Public-Private Mix") OR (Financ* OR Spending OR Expense* OR Expenditures* OR Costs OR Spending OR Payment* OR Coverage OR Deliver* OR Provider* OR Regulation OR Access OR Utilization OR Provision OR "Health Consortia" OR "Healthcare Financing" OR "Health Insurance")) AND ("Latin America" OR "Latinoamérica" OR "América Latina" OR Brasil* OR Brazil OR Equador OR Ecuador OR México OR Mexico OR Chile OR Colômbia OR Colombia OR Peru OR Perú OR Peru* OR Argentin*) AND ("Health inequalit*" OR "Health inequit*" OR "Health disparit*" OR "Health assymetr*" OR "Health Status Disparities" OR "Socioeconomic Disparities in Health" OR "Social Determinants of Health") | |
| **Fields used in the database:**   - All Fields; - Period: 2000-2024 | |
| **Scopus** |  |
| **Strategy:** ("Health system" OR "Health systems" OR "Health care system" OR "Health care systems" OR "Health policy" OR "Health polic*" OR "Health reform*" OR "Unified Health System" OR SUS OR "National Health Systems") AND (("Public private" OR Public-private OR "Public and private" OR "Private sector" OR Privatization OR "Private plans" OR "Private insurance" OR "Public Sector" OR "Public-Private Sector Partnerships" OR "Public-Private Mix") OR (Financ* OR Spending OR Expense* OR Expenditures* OR Costs OR Spending OR Payment* OR Coverage OR Deliver* OR Provider* OR Regulation OR Access OR Utilization OR Provision OR "Health Consortia" OR "Healthcare Financing" OR "Health Insurance")) AND ("Latin America" OR "Latinoamérica" OR "América Latina" OR Brasil* OR Brazil OR Equador OR Ecuador OR México OR Mexico OR Chile OR Colômbia OR Colombia OR Peru OR Perú OR Peru* OR Argentin*) AND ("Health inequalit*" OR "Health inequit*" OR "Health disparit*" OR "Health assymetr*" OR "Health Status Disparities" OR "Socioeconomic Disparities in Health" OR "Social Determinants of Health") | |
| **Fields used in the database:**   - Title; - Subject; - Abstract; - Period: 2000-2024 | |
| **Web of Science** |  |
| **Strategy:** (ALL=(("Health system" OR "Health systems" OR "Health care system" OR "Health care systems" OR "Health policy" OR "Health polic*" OR "Health reform*" OR "Unified Health System" OR SUS OR "National Health Systems") AND (("Public private" OR Public-private OR "Public and private" OR "Private sector" OR Privatization OR "Private plans" OR "Private insurance" OR "Public Sector" OR "Public-Private Sector Partnerships" OR "Public-Private Mix") OR (Financ* OR Spending OR Expense* OR Expenditures* OR Costs OR Spending OR Payment* OR Coverage OR Deliver* OR Provider* OR Regulation OR Access OR Utilization OR Provision OR "Health Consortia" OR "Healthcare Financing" OR "Health Insurance")) AND ("Health inequalit*" OR "Health inequit*" OR "Health disparit*" OR "Health assymetr*" OR "Health Status Disparities" OR "Socioeconomic Disparities in Health" OR "Social Determinants of Health"))) AND ALL=(("Latin America" OR "Latinoamérica" OR "América Latina" OR Brasil* OR Brazil OR Equador OR Ecuador OR México OR Mexico OR Chile OR Colômbia OR Colombia OR Peru OR Perú OR Peru* OR Argentin*) ) | |
| **Fields used in the database:**   - All Fields; - Period: 2000-2024 | |

Source: Elaborated by the authors with the assistance of a librarian..
