# Supplementary material for: Public-private mix in health systems and repercussions for health inequalities in Latin American countries: A scoping review protocol
Source: PLoS One. 2026 Feb 19;21(2):e0305437. doi: 10.1371/journal.pone.0305437 (PMC12919785; doi:10.1371/journal.pone.0305437)
Supplement: S2 Appendix — (DOCX) [file pone.0305437.s002.docx]

**S2 Appendix** **2**– **Data extraction instrument.**

| ID | Article identification code with letter and number in the database. |
| --- | --- |
| Publication year | Year the article was published. |
| Author’s | List the authors of the article. |
| Title | Publication title. |
| DOI/URL | Electronic address of the article. |
| Abstract | Article abstract. |
| Journal | Journal of the publication. |
| Language | Language of the publication. |
| Approach/type of study | Approach or type of study declared by the authors in the publication. |
| Country | Country studied in the article. |
| Public-private configuration and repercussions for health inequalities (and response to covid-19) | Results of the article that address the central themes. |
| Central theme | Central themes addressed in the publication (financing, coverage, provision). |
| Observations | Comments on publication or extraction. |

Source: Elaborated by the authors.
