# Supplementary material for: Public-private mix in health systems and repercussions for health inequalities in Latin American countries: A scoping review protocol
Source: PLoS One. 2026 Feb 19;21(2):e0305437. doi: 10.1371/journal.pone.0305437 (PMC12919785; doi:10.1371/journal.pone.0305437)
Supplement: S3 Appendix — (DOCX) [file pone.0305437.s003.docx]

**S3 Appendix** **3 – Timeline for the review.**

|  | Month 1 | Month 2 | Month 3 | Month 4 | Month 5 | Month 6 | Month 7 |
| --- | --- | --- | --- | --- | --- | --- | --- |
| Searching the databases |  |  |  |  |  |  |  |
| Data extraction |  |  |  |  |  |  |  |
| Double-blind peer review |  |  |  |  |  |  |  |
| Reading titles and abstracts |  |  |  |  |  |  |  |
| Read the full articles |  |  |  |  |  |  |  |
| Review by the third reviewer. |  |  |  |  |  |  |  |
| Final selection of the articles |  |  |  |  |  |  |  |
| Complete reading of the documents included |  |  |  |  |  |  |  |
| Extracting and analyzing articles |  |  |  |  |  |  |  |
| Expert consultation |  |  |  |  |  |  |  |
| Writing the review article |  |  |  |  |  |  |  |

Source: Elaborated by the authors.
